# Curcumin piperidone derivatives induce anti-proliferative and anti-migratory effects in LN-18 human glioblastoma cells

**DOI:** 10.1038/s41598-022-16274-4

**Published:** 2022-07-30

**Authors:** Nur Syahirah Che Razali, Kok Wai Lam, Nor Fadilah Rajab, A. Rahman A. Jamal, Nurul Farahana Kamaluddin, Kok Meng Chan

**Affiliations:** 1 Center for Toxicology and Health Risk Studies, Faculty of Health Sciences, Universiti Kebangsaan Malaysia, 50300 Kuala Lumpur, Malaysia; 2 Center for Healthy Ageing and Wellness, Faculty of Health Sciences, Universiti Kebangsaan Malaysia, 50300 Kuala Lumpur, Malaysia; 3 Center for Drug and Herbal Development, Faculty of Pharmacy, Universiti Kebangsaan Malaysia, 50300 Kuala Lumpur, Malaysia; 4 UKM Medical Molecular Biology Institute, UKM Medical Centre, 56000 Cheras, Malaysia; 5 Institute for Environmental and Development, UKM, 43600 Bangi, Selangor Malaysia

**Keywords:** CNS cancer, Apoptosis, Cell migration, Cell invasion, Toxicology, Drug development

## Abstract

Curcumin has demonstrated potential cytotoxicity across various cell lines despite its poor bioavailability and rapid metabolism. Therefore, our group have synthesized curcuminoid analogues with piperidone derivatives, FLDP-5 and FLDP-8 to overcome these limitations. In this study, the analogues were assessed on LN-18 human glioblastoma cells in comparison to curcumin. Results from cytotoxicity assessment showed that FLDP-5 and FLDP-8 curcuminoid analogues caused death in LN-18 cells in a concentration-dependent manner after 24-h treatment with much lower IC_50_ values of 2.5 µM and 4 µM respectively, which were more potent compared to curcumin with IC_50_ of 31 µM. Moreover, a significant increase (*p* < 0.05) in the level of superoxide anion and hydrogen peroxide upon 2-h and 6-h treatment confirmed the oxidative stress involvement in the cell death process induced by these analogues. These analogues also showed potent anti-migratory effects through inhibition of LN-18 cells’ migration and invasion. In addition, cell cycle analysis showed that these analogues are capable of inducing significant (*p* < 0.05) S-phase cell cycle arrest during the 24-h treatment as compared to untreated, which explained the reduced proliferation indicated by MTT assay. In conclusion, these curcuminoid analogues exhibit potent anti-cancer effects with anti-proliferative and anti-migratory properties towards LN-18 cells as compared to curcumin.

## Introduction

Glioblastoma multiforme (GBM) is a grade IV astrocytoma characterized by rapid infiltrating growth and is the most common and aggressive malignant primary brain tumor in humans^1,2^. GBM patients have a poor prognosis whereby most of the patients hardly survive for more than one year; with less than 3% of patients surviving for more than 5 years after diagnosis^3^. This is mainly due to the extensive heterogeneity at the cellular and molecular levels of the tumour and also the issue of the resistance towards the chemotherapeutic drug, temozolomide^4–6^. Several studies had reported that glioblastoma cells, including LN-18 cells, were resistant to temozolomide via upregulation of methylguanine methyltransferase (MGMT) enzyme and deficiency in mismatch repair (MMR) mechanism^7,8^. Due to the high frequency of drug resistance, GBM remains challenging to deal with the drug-mediated therapy. As a consequence of the poor efficacy of crossing the blood–brain barrier (BBB), most chemotherapy medicines, such as doxorubicin and cisplatin, have failed to treat this tumour^9,10^. Therefore, finding novel approaches is an urgent priority for the improvement of patients’ prognosis. With the aim for the drug-mediated therapy to pursue, identifying a potent compound that could defeat the resistance of GBM in addition to BBB-crossing ability is desperately needed.

Turmeric, a rich source of curcumin, has been used for centuries in traditional remedies to treat a variety of diseases^11,12^. With the advancement in technology over the years, the biological activities of curcumin and its molecular targets have been identified. Curcumin has demonstrated a wide range of biological activities, including anti-inflammatory, cytotoxicity and apoptosis induction on several cancer cell lines^12–17^. Numerous molecular targets for curcumin, for instance, tumour suppressor proteins (p53 & p21), oncoproteins (C-Myc, cyclin D1) and antiapoptotic proteins (survivin & Bcl-2), have been identified and reported crucial in curcumin-induced apoptosis^18–20^. Despite this, curcumin has its own drawbacks, whereby clinical trials and animal studies on curcumin showed that this compound has poor bioavailability and weak pharmacokinetic profile, rendering it a poor drug candidate. The poor bioavailability is mainly contributed by poor absorption as well as rapid metabolism and elimination via sequential reduction and glucuronidation by the body^21–23^.

**Figure 1:**
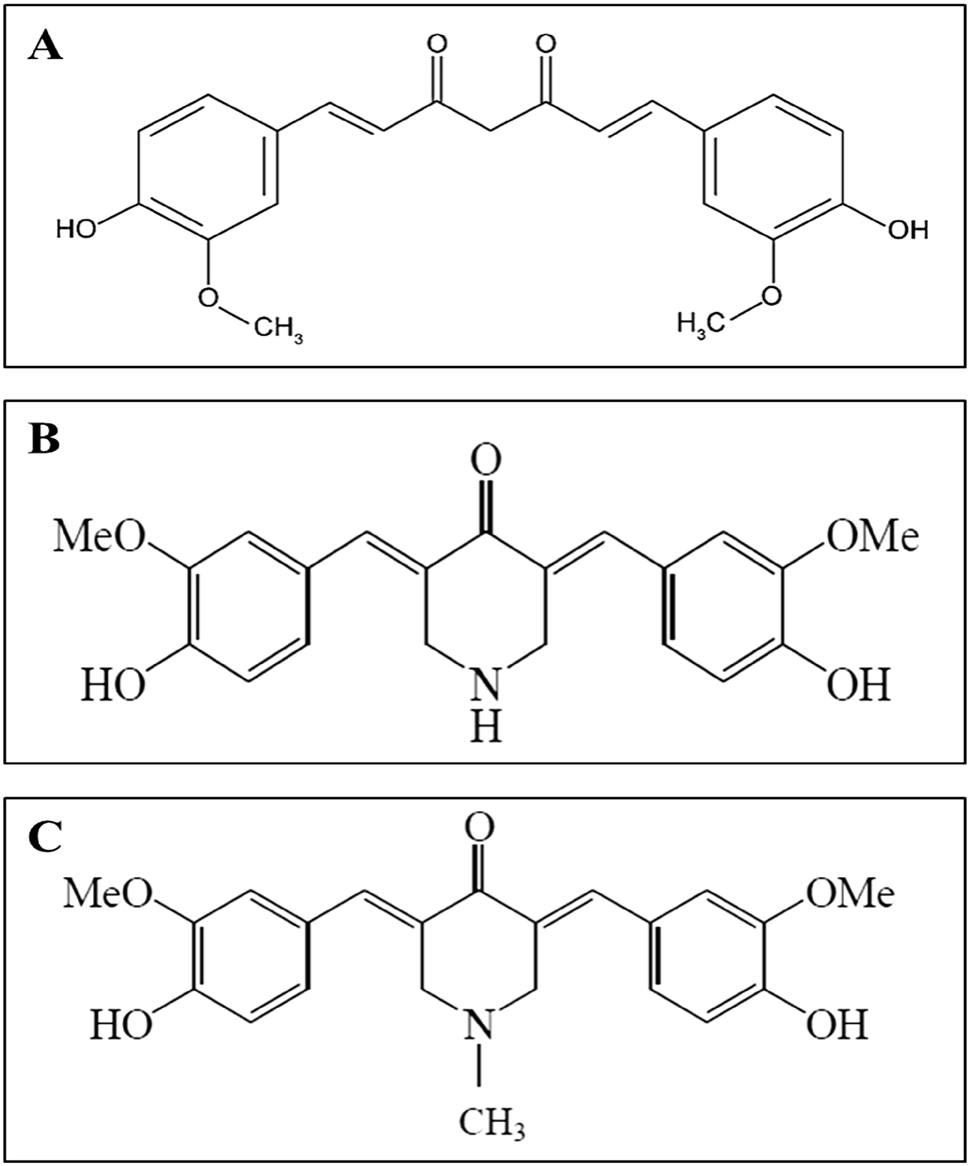
Chemical structures of test compounds in this study (**A**) Chemical structure of curcumin (**B**) Chemical structure of curcuminoid analogue FLDP-5 with molecular name 4-Peperidinone,3,5-bis[(4-hydroxy-3-methoxyphenyl) methylene]-,(3E,5E) (Molecular weight: 367.40 g/mol) (**C**) Chemical structure of curcuminoid analogue FLDP-8 with molecular name 4-Peperidinone,3,5-bis[(4-hydroxy-3-methoxyphenyl)methylene]-1-Methyl(3E,5E) (Molecular weight: 381.42 g/mol).

Chemical synthesis and modification are commonly used to produce new derivatives of chemotherapeutic drugs with improved efficacy, bioavailability and selectivity^24,25^. Hence, our group have synthesized two curcuminoid analogues with two piperidone derivatives, namely FLDP-5 and FLDP-8 (Fig. 1). Previous study has found that piperidone could increased the absoption of curcumin which contribute tonhigherb activitybof this compounds. In the present study, we have compared the effectiveness of these curcuminoid analogues and natural curcumin on GBM cell lines derived from human (LN-18). This study demonstrated that FLDP-5 and FLDP-8 curcuminoid analogues exhibited highly potent tumour-suppressive effects with anti-proliferative and anti-migratory activities on LN-18 cells compared to curcumin. A prior study found that piperidone increased curcumin absorption^26,27^. Although we did not examine the absorption of the curcumin-related compounds containing piperidone derivatives in this work, it is plausible that improved absorption contributes to higher activity of these compounds. Further research is needed to identify the cellular absorption of these compounds.

## Results

### Curcuminoid analogues (FLDP-5 and FLDP-8) induced cytotoxicity on LN-18 human GBM cells and HBEC-5i cells

**Figure 2:**
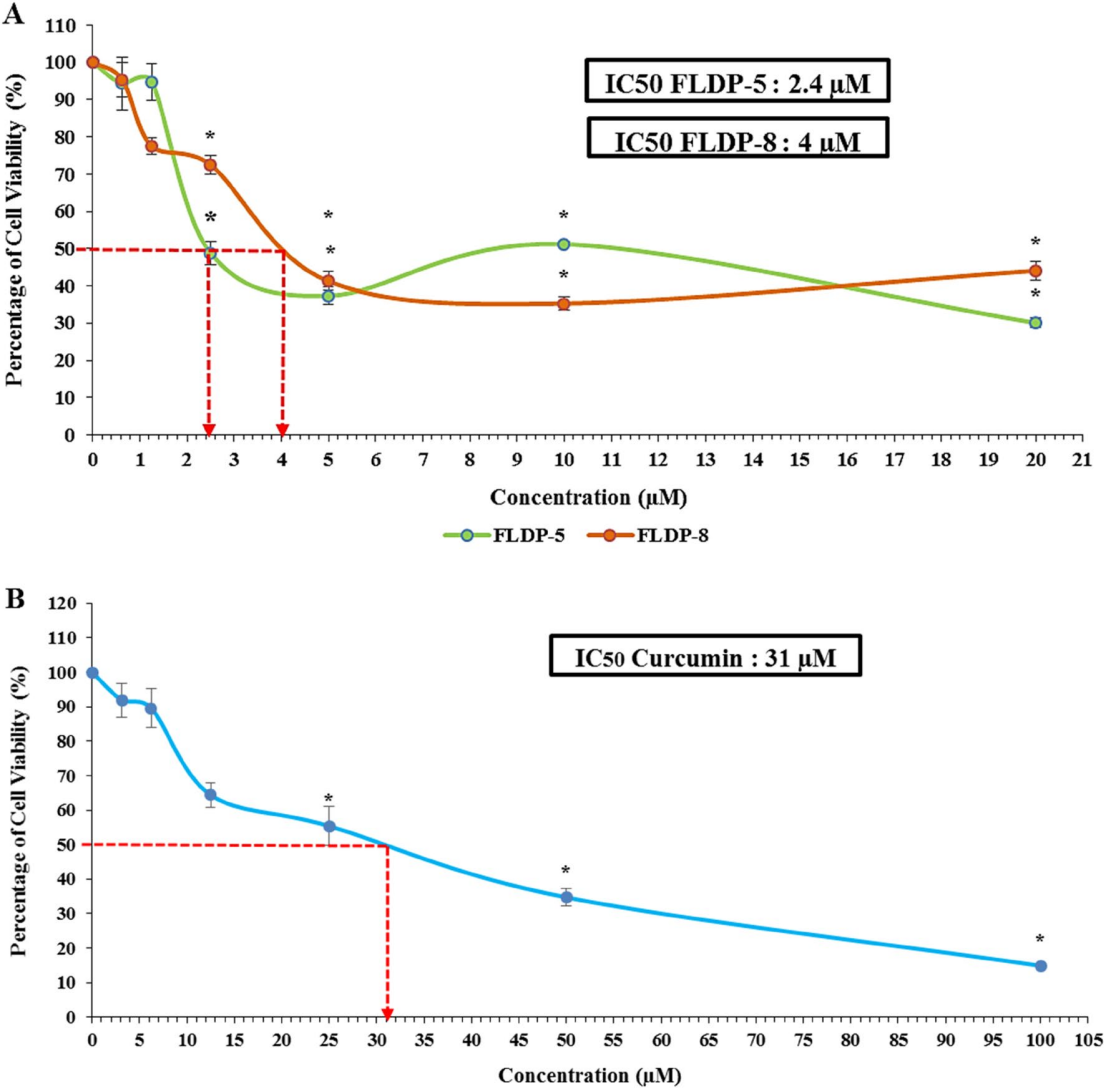
The cytotoxicity assessment of curcuminoid analogues (FLDP-5 and FLDP-8) and curcumin on LN-18 cells. (**A**) Cytotoxicity of FLDP-5 and FLDP-8 curcuminoid analogues treated LN-18 cells with concentrations from 0.625 μM till 20 μM was observed after 24-h treatment. IC_50_ values of 2.4 μM and 4 μM were observed respectively in FLDP-5 curcuminoid analogues and FLDP-8. (**B**) Cytotoxicity of curcumin-treated LN-18 cells with concentrations from 3.125 μM till 100 μM was observed after 24-h treatment. An IC_50_ value of 31 μM was observed. Each data point was obtained from three independent experimental replicates and expressed as mean ± SEM of percentage of cell viability. *p < 0.05 against negative control (untreated cell).

**Figure 3:**
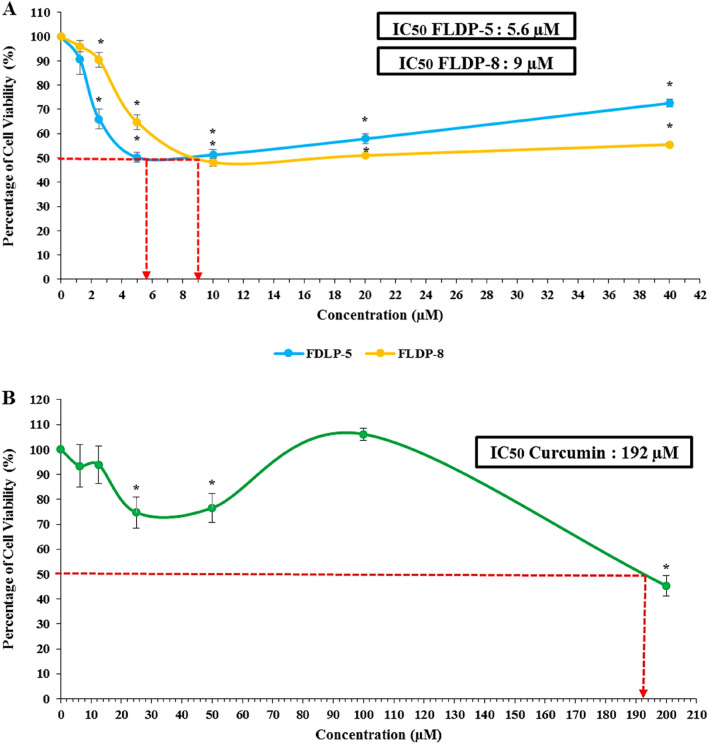
The cytotoxicity assessment of curcuminoid analogues (FLDP-5 and FLDP-8) and curcumin on HBEC-5i cells. (**A**) Cytotoxicity of FLDP-5 and FLDP-8 curcuminoid analogues treated HBEC-5i cells with concentrations from 1.25 μM till 40 μM was observed after 24-h treatment. IC_50_ values of 5.6 μM and 9 μM were observed respectively in FLDP-5 and FLDP-8 curcuminoid analogues. (**B**) Cytotoxicity of curcumin-treated HBEC-5i cells with concentrations from 6.25 μM till 200 μM was observed after 24-h treatment. An IC_50_ value of 192 μM was observed. Each data point was obtained from three independent experimental replicates and expressed as mean ± SEM of percentage of cell viability. *p < 0.05 against negative control (untreated cell).

**Figure 4:**
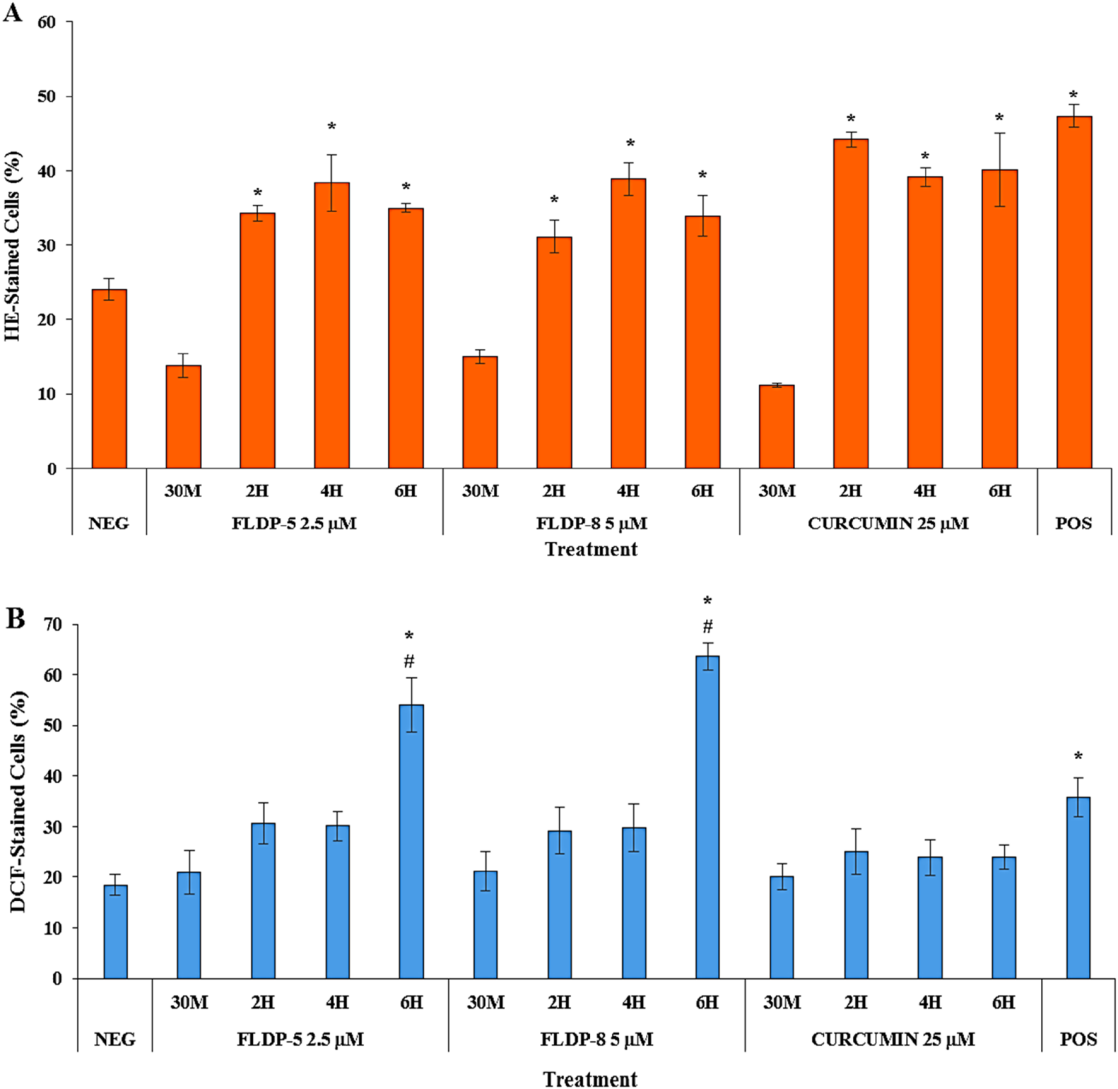
ROS production assessment in LN-18 cells. (**A**) Flow cytometric analysis of superoxide level using HE staining. (**B**) Flow cytometric analysis of hydrogen peroxide level using DCFH-DA staining. Cells were treated respectively with IC_50_ values of FLDP-5, FLDP-8 and curcumin at different time-points ranging from 30 min until 6-h. Both assays used HQ treatment at 12.5 μM for 6-h as positive control (POS). Each data point was obtained from three independent experimental replicates and expressed as mean ± SEM of HE- or DCF-stained cells (%). *p < 0.05 against negative control, NEG and # *p* < 0.05 against curcumin.

The cytotoxic effects of curcuminoid analogues (FLDP-5 and FLDP-8) and curcumin were determined using MTT cytotoxicity assay. The results showed that the curcuminoid analogues, FLDP-5 and FLDP-8, including curcumin, induced cytotoxicity in LN-18 cells in a concentration-dependent manner after 24-h treatment. Interestingly, the IC_50_ values observed for the FLDP-5 and FLDP-8 curcuminoid analogues were 2.4 µM (Fig. 2A) and 4 µM (Fig. 2A), respectively, which were more potent in comparison to curcumin with an IC_50_ value of 31 µM (Fig. 2B). Evaluation of FLDP-5 and FLDP-8 curcuminoid analogues toxicity on the non-cancerous HBEC-5i cell line, showed that much higher doses were required to cause HBEC-5i cell viability to decrease by 50% compared to the LN-18 cancer cell lines. The IC_50_ values of curcuminoid analogues (FLDP-5 and FLDP-8) and curcumin in HBEC-5i were determined as 5.6 ± 0.5 (Fig. 3A), 9 ± 0.66 (Fig. 3A), and 192 ± 4.67 (Fig. 3B), respectively. As shown previously^28,29^, the IC_50_ values were used to calculate the selective index, SI (Table 1), a baseline used to assess the selective toxicity of the analogues and curcumin towards cancerous cells over normal cells. It was noteworthy that the SI for both analogues in HBEC-5i were 2.33-fold and 2.25-fold higher (Table 1) respectively compared to the baseline (100) showing the selectivity of these analogues towards cancerous cells than normal cells. Hydroquinone (HQ) is used as the positive control in this study, where the IC_50_ value is 10 µM following 24-h treatment in LN-18 cells.

**Table 1:** IC_50_ of the compounds in LN-18 cells and HBEC-5i cells including the selectivity index (SI).

Compounds	IC_50_ (µM) ± S.E.M		SI
	LN-18 cells	HBEC-5i cells	
FLDP-5	2.4 ± 0.12	5.6 ± 0.5	233
FLDP-8	4 ± 0.12	9 ± 0.66	225
Curcumin	31 ± 5.46	192 ± 4.67	619

### FLDP-5 and FLDP-8 curcuminoid analogues were predicted to be BBB permeable

The probability of the analogues penetrating the BBB were first estimated by an online platform: AlzPlatform with cloud computing and sourcing functions. The analysis showed that FLDP-5 and FLDP-8 curcuminoid analogues including curcumin had positive BBB scores (Supplementary Fig. S1), implying that these analogues and curcumin are permeable to the BBB. We further confirmed this prediction using another online predictor which is ADMETlab 2.0 that were usually used in predicting the pharmacokinetic properties of compounds such as absorption, distribution, metabolism, excretion, and toxicity (ADMET). The results from distribution section of the reports stated the similar predictions with the previous predictor which showed that curcuminoid analogues (FLDP-5 and FLDP-8) and curcumin were capable to cross the BBB with the output value of the probability of being BBB + were 0.029, 0.38 and 0.155 (Supplementary Table S1) respectively. It is depicted that FLDP-5 curcuminoid analogue and curcumin showed excellent output values of the probability of being BBB + compared to FLDP-8 curcuminoid analogue with medium output value of the probability of being BBB + .

### FLDP-5 and FLDP-8 curcuminoid analogues induced both superoxide and hydrogen peroxide in LN-18 cell death

Intracellular ROS, specifically superoxide anion and hydrogen peroxide, were assessed using HE and DCFH-DA staining and detected through flow cytometer. The detection was performed to determine the ROS involvement in inducing oxidative stress and consecutively cell death in LN-18-treated cells. Our study demonstrated curcuminoid analogues (FLDP-5 and FLDP-8), and curcumin caused a significant elevated level of superoxide anion as compared to negative control cells with respective 1.42-fold, 1.26-fold and 1.83-fold increase at 2-h time-point treatment and persisted up to 6-h (Fig. 4A). Interestingly, Fig. 4B showed that FLDP-5 and FLDP-8 curcuminoid analogues were also able to induce a significant level of hydrogen peroxide with 2.93-fold and 3.45-fold increase respectively at 6-h time-point treatment in comparison to negative control cells. Consequently, these analogues showed a significant difference in which different reactions compared to parent compound curcumin as hydrogen peroxide level was not induced in curcumin-treated LN-18 cells.

### Curcuminoid analogues (FLDP-5 and FLDP-8) induced higher severity of DNA damage compared to curcumin-treated LN-18 cells

**Figure 5:**
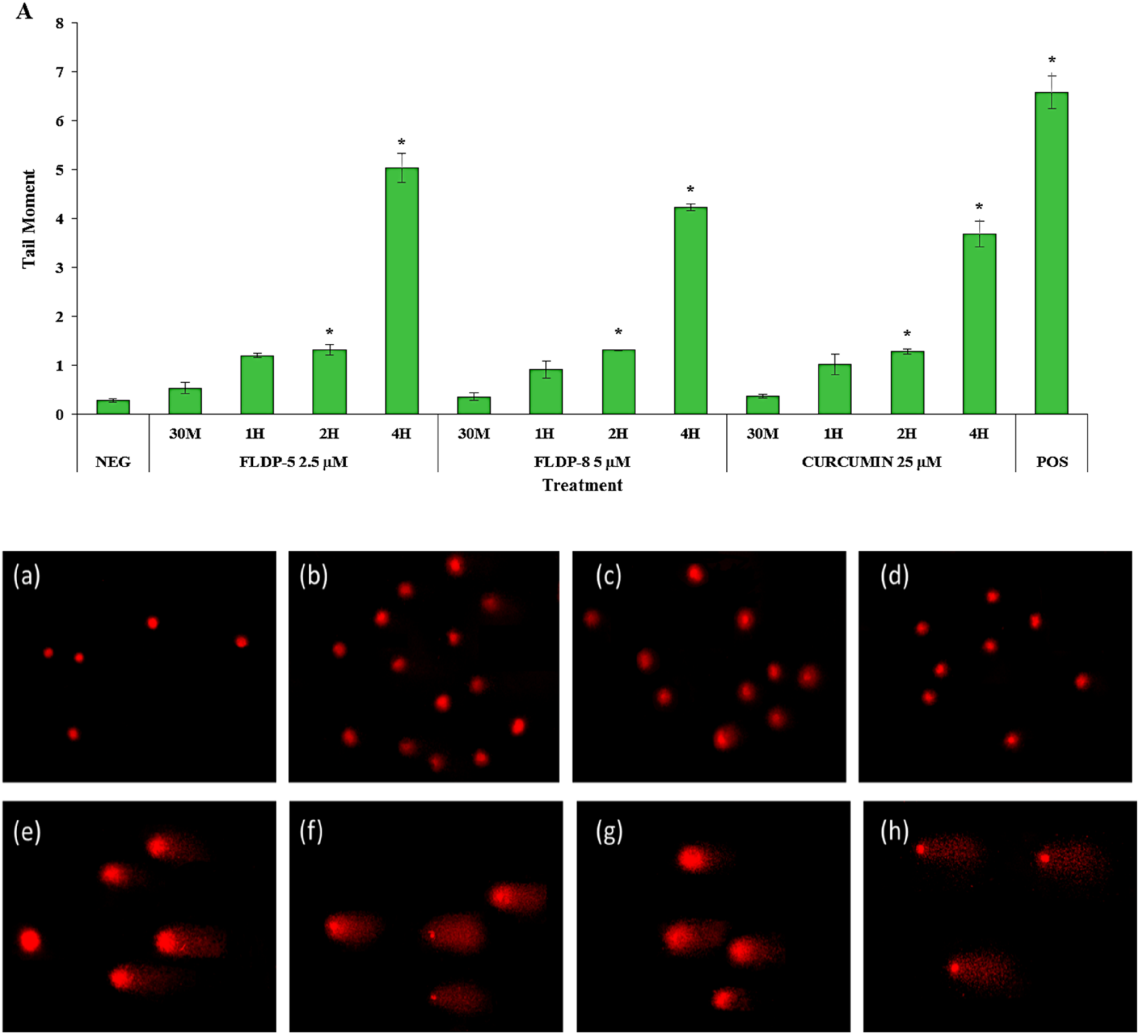
Assessment of DNA damage in LN-18 using alkaline comet assay. (**A**) DNA damage expressed as tail moment in cells treated respectively with IC_50_ values of FLDP-5, FLDP-8 and curcumin at different time-points ranging from 30 min until 6-h. Fluorescence microscopic images stained with EtBr stain of untreated cells (a), cells treated with FLDP-5 at 2.5 μM for 4-h (b), FLDP-8 at 5 μM for 4-h (c), curcumin at 25 μM for 4-h (d), FLDP-5 at 2.5 μM for 6-h (e), FLDP-8 at 5 μM for 6-h (f), curcumin at 25 μM for 6-h (g) and positive control (h). Each data was obtained from three independent experimental replicates and each data point in (**A**) was expressed as mean ± SEM of tail moment. *p < 0.05 against negative control, NEG and # *p* < 0.05 against curcumin.

In this study, the occurrence of DNA damage induced by the curcuminoid analogues and curcumin on LN-18 cells were assessed using alkaline comet assay. The scoring for DNA damage was based on the length of the formed tail, which represents the migration of DNA with strand breakage during electrophoresis following treatment. The images were captured using a fluorescence microscope and depicted in Fig. 5. In the negative control group, only a small number of cells showed DNA damage with tail moment of 0.28 ± 0.03. However, the DNA damage in all treated compounds progressively increased in a time-dependent manner following respective treatments using IC_50_ values (Fig. 5A). In contrast to the negative control, severe damages at 6-h time-point treatment with a significantly higher tail moment up to 192-fold, 211-fold and 122-fold increase respectively were observed in curcuminoid analogues (FLDP-5 and FLDP-8) and curcumin. Curcuminoid analogues (FLDP-5 and FLDP-8) were also observed to have a significantly higher tail moment (*p* < 0.05) with respective values of 53.97 ± 4.54 and 59.23 ± 4.71 compared to parent compound curcumin with tail moment of 34.35 ± 4.9 following 6-h treatment.

### Curcuminoid analogues (FLDP-5 and FLDP-8) and curcumin potentiated anti-migration effects in LN-18 cells

**Figure 6:**
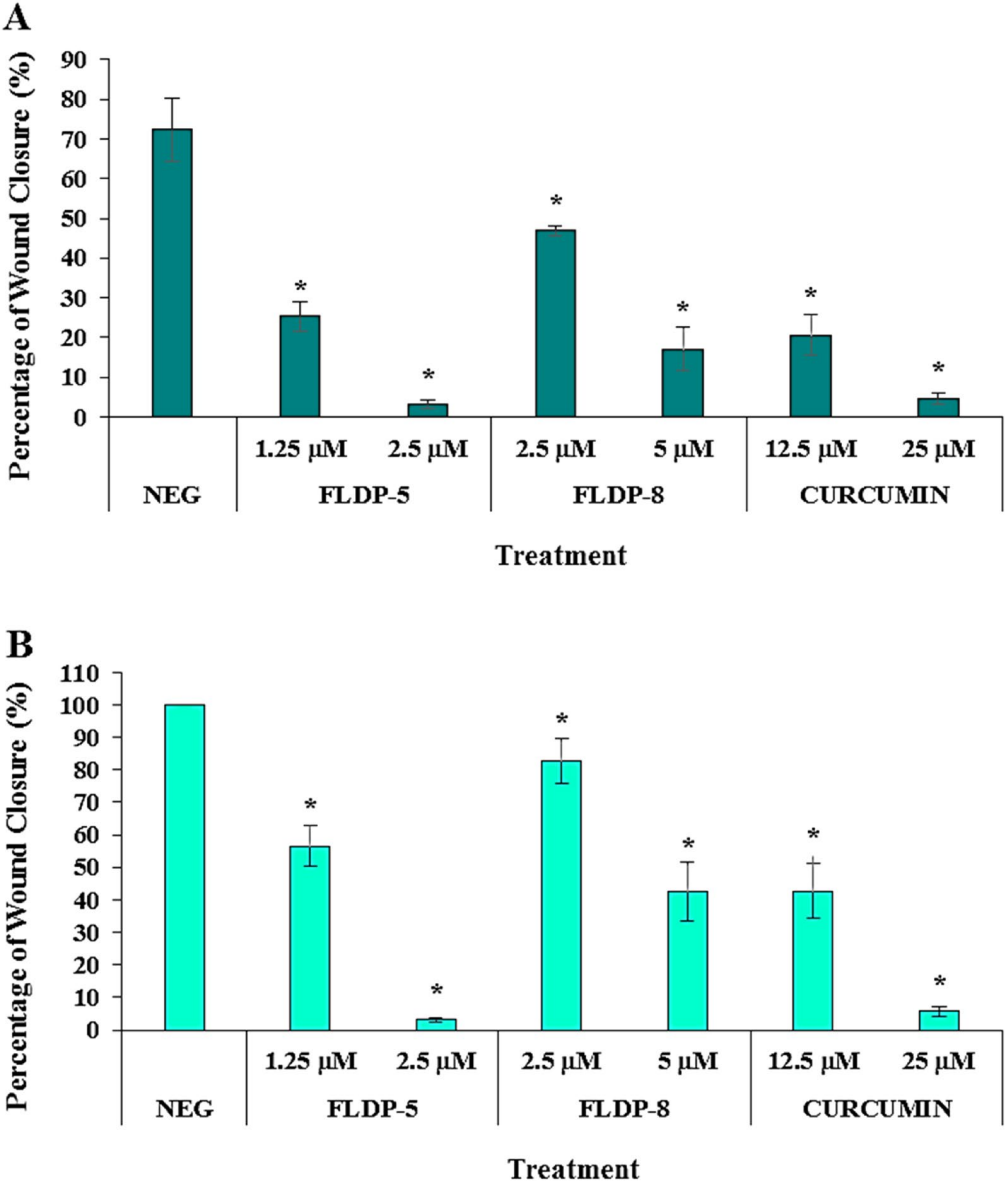

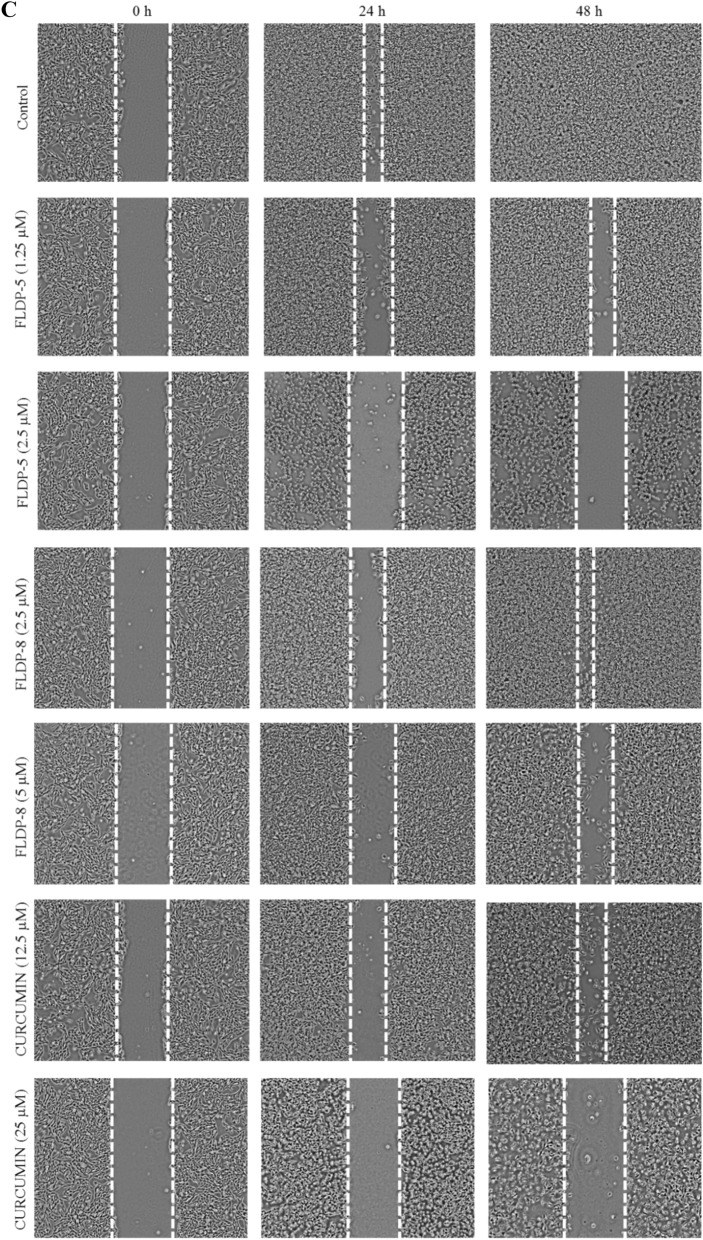
Assessment of cell migration in LN-18 cells using scratch/wound-healing assay. (**A**) LN-18 cells were treated respectively with IC_50_ and IC_25_ of FLDP-5, FLDP-8 curcuminoid analogues and curcumin at 24-h and the percentage of wound closure was measured. (**B**) LN-18 cells were treated respectively with IC_50_ and IC_25_ of FLDP-5, FLDP-8 curcuminoid analogues and curcumin at 48-h and the percentage of wound closure was measured. (**C**) Microscopic images of untreated cells, cells treated with FLDP-5 curcuminoid analogue (1.25 μM and 2.5 μM), FLDP-8 curcuminoid analogue (2.5 μM and 5 μM) and curcumin (12.5 μM and 25 μM) for 0 h, 24-h and 48-h. Each data was obtained from three independent experimental replicates and each data point in (**A**) and (**B**) was expressed as mean ± SEM of wound closure percentage. *p < 0.05 against negative control.

The anti-migratory effects of curcuminoid analogues (FLDP-5 and FLDP-8) and curcumin on LN-18 cells were investigated through wound scratch assay. The migration rate of treated LN-18 cells with respective compounds was calculated based on the closure of the wound/ scratch on the monolayer LN-18 cells. The images of the closure for each treatment were captured using a camera attached an inverted microscope and represented in Fig. 6C. It could be observed that treatment with IC_50_ and IC_25_ of curcuminoid analogues (FLDP-5 and FLDP-8) and curcumin showed a significant concentration-dependent decrease in the percentage of wound closure compared to the untreated group after 24-h and 48-h incubation time (Fig. 6A and Fig. 6B). The percentage of wound closure in the untreated group reached 100%, as indicated in Fig. 6B and Fig. 6C after 48-h incubation time, indicating the high rate of motility and rapid growth characteristics of LN-18 glioblastoma cells^30^. Our results demonstrated that curcumin and FLDP-5 curcuminoid analogue showed a higher potential in inhibiting the migration of LN-18 cells when treated with values of IC_25_ and IC_50_ compared to FLDP-8 curcuminoid analogue. After 48-h treatment, percentage of wound closure of curcumin and FLDP-5 curcuminoid analogue when treated with IC_25_ concentration is 42.74% ± 8.32 and 56.43% ± 6.28 respectively, which were lower than the percentage of wound closure of FLDP-8 curcuminoid analogue treated with IC_25_ concentration, which was 82.69% ± 7.02 (Fig. 6B). Similar to the previous one, the percentage of wound closure when treated with IC_50_ values of curcumin and FLDP-5 curcuminoid analogue after 48-h were also much lower than FLDP-8 curcuminoid analogue with the percentage of wound closure of 5.69% ± 1.56, 3.17% ± 0.71 and 42.6% ± 9.1 respectively (Fig. 6B).

### FLDP-5 and FLDP-8 curcuminoid analogues and curcumin inhibited the invasion of LN-18 cells

**Figure 7:**
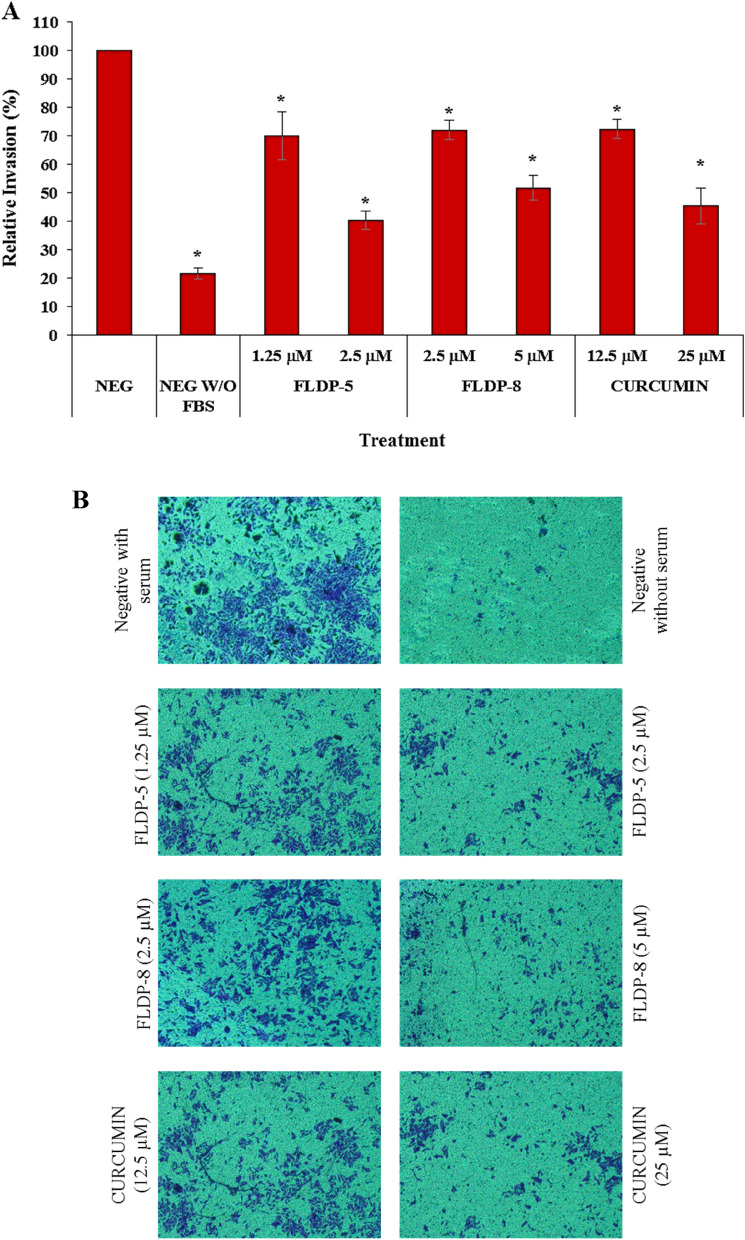
Assessment of cell invasion in LN-18 cells using Boyden chamber transwell assay. (**A**) LN-18 cells were treated respectively with IC_50_ and IC_25_ of FLDP-5, FLDP-8 curcuminoid analogues and curcumin at 24-h and the number of invaded of cells was measured at 560 nm. (**B**) Microscopic images of untreated cells with serum, untreated cells without serum, cells treated with FLDP-5 curcuminoid analogue (1.25 μM and 2.5 μM), FLDP-8 curcuminoid analogue (2.5 μM and 5 μM) and curcumin (12.5 μM and 25 μM) for 24-h. Each data was obtained from three independent experimental replicates and each data point was expressed as mean ± SEM of percentage of relative invasion. *p < 0.05 against negative control, NEG.

To study the effects of curcuminoid analogues (FLDP-5 and FLDP-8) and curcumin on the invasive behaviours of LN-18 cells, we performed a transwell invasion assay with modified Boyden chambers. Our findings were consistent in corresponding to the wound healing experiments, with significant decline in the invasiveness of cells following the treatment of curcuminoid analogues and curcumin. All treatment compounds were able to reduce the percentage of relative invasion in LN-18 cells in a significant dose-dependent manner compared to the untreated group after 24-h treatment (Fig. 7A). Treatment of FLDP-5 curcuminoid analogue at IC_50_ concentration showed the lowest relative invasion at 2.48-fold decrease with a percentage of 40.32% ± 3.14. The untreated group without chemo-attractant (serum: FBS) was used as an indicator that invasive properties of LN-18 were affected through the absence or presence of chemo-attractant (Fig. 7A and Fig. 7B).

### FLDP-5 and FLDP-8 curcuminoid analogues induced cell cycle arrest in LN-18 treated cells

**Figure 8:**
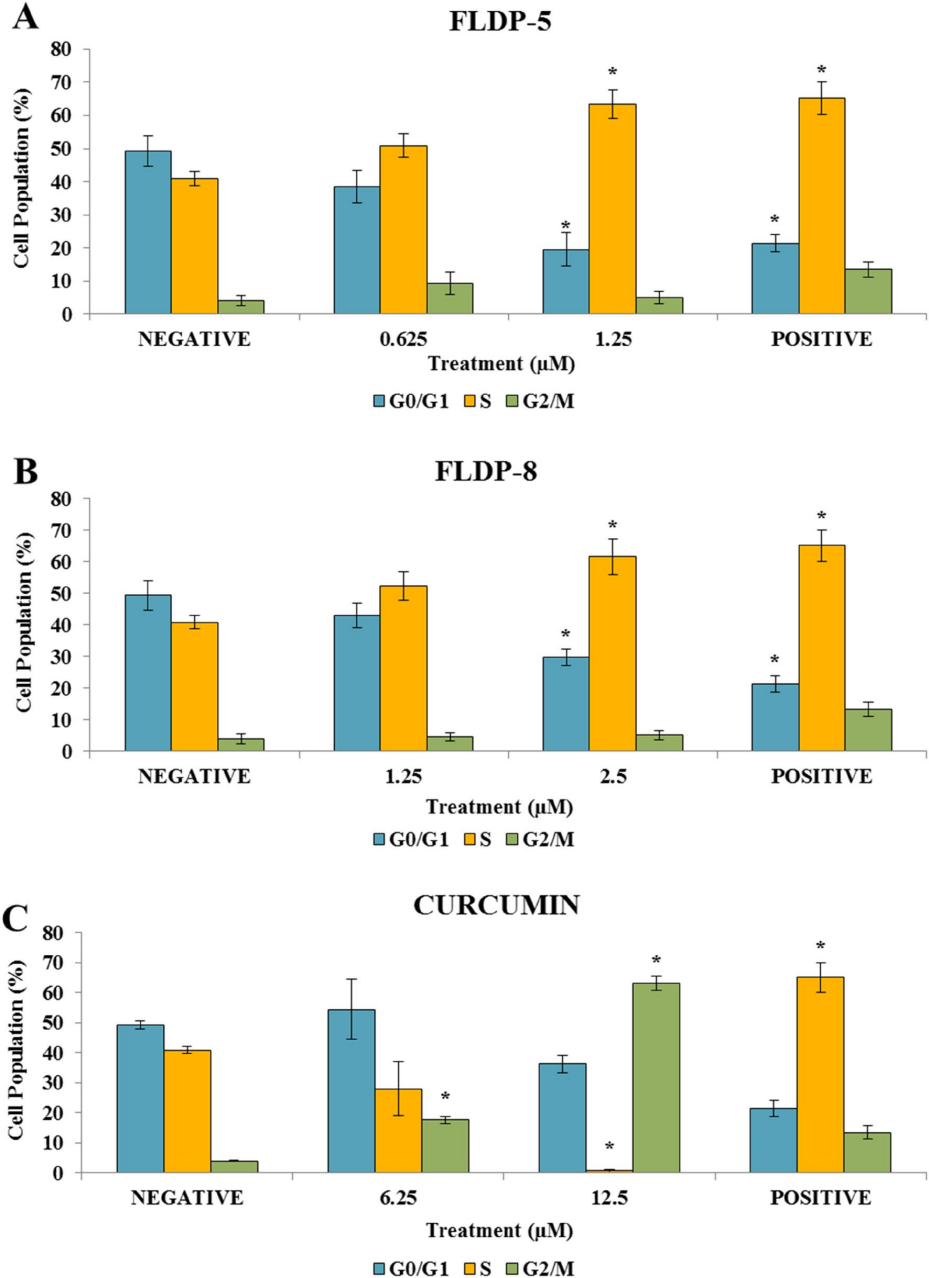
Assessment of cell cycle arrest in LN-18 using PI/RNase staining. (**A**) LN-18 cells were treated respectively with IC_25_ and IC_12.5_ of FLDP-5 at 24-h and the percentage cell population in each phase is measured. (**B**) LN-18 cells were treated respectively with IC_25_ and IC_12.5_ of FLDP-8 at 24-h and the percentage cell population in each phase is measured. (**C**) LN-18 cells were treated respectively with IC_25_ and IC_12.5_ of curcumin at 24-h and the percentage cell population in each phase is measured. Each data was obtained from three independent experimental replicates and each data point in (**A**), (**B**) and (**C**) was expressed as mean ± SEM of cell population percentage. *p < 0.05 against negative control.

Cell cycle analysis was conducted to determine the involvement of cell cycle arrest in FLDP-5 and FLDP-8 curcuminoid analogues mechanism of action on LN-18 cells. Flow cytometric assessment was performed following 24-h treatment using IC_25_ and IC_12.5_ of all compounds to detect the cell population of each phase. Our study demonstrated that FLDP-5 and FLDP-8 curcuminoid analogues were able to induce arrest in S phase in a concentration-dependent manner, but a significant arrest in S phase with 1.5-fold increase for both analogues were observed when LN-18 cells when treated with IC_25_ values by accumulating 63.38% ± 4.42 and 61.59% ± 5.66 respectively (Fig. 8A and 8B). Contradictory results were observed in curcumin-treated LN-18 cells where the cell cycle inhibition occurred significantly at the G_2_/M phase (Fig. 8C). Curcumin induced arrest in the G_2_/M phase in a concentration-dependent manner and reached significant comparison with the untreated group after treatment of IC_25_ value with a cell population of 62.99% ± 2.38.

## Discussion

The available standard treatments for GBM have been found to be ineffective due to the inherent resistance of GBM cells to radiotherapy and chemotherapy, with the addition to the invasive behaviour of GBM cells, causing the effectiveness of surgery to be limited^31,32^. Moreover, because of the high frequency of drug resistance, GBM remains challenging to deal with through the drug-mediated therapy. Due to the poor efficacy of crossing the BBB, most chemotherapy medicines, such as doxorubicin and cisplatin, have failed to treat this tumour^9,10^. Therefore, finding novel approaches is an urgent priority for the improvement of patients’ prognosis. With the aim for the drug-mediated therapy to pursue, identifying a potent compound that could defeat the resistance of GBM in addition to BBB-crossing ability is desperately needed. Recently, natural polyphenol curcumin has been found to be able to attenuate GBM growth, proliferation, and metastasis in vitro and in vivo models of glioma^31,33^. However, the major concern regarding utilizing curcumin in treating GBM is its problems which having poor solubility, rapid degradation, and limited bioavailability, as reported by few researchers. These drawbacks may limit the efficacy of curcumin therapy in GBM^34,35^. Therefore, in the present study, we have compared the effiectiveness of newly synthesized curcumin derivatives, namely FLDP-5 and FLDP-8 curcuminoid analogues and natural curcumin on GBM cell lines derived from human (LN-18). This study demonstrated that FLDP-5 and FLDP-8 curcuminoid analogues exhibit a highly potent tumour-suppressive effect on LN-18 human GBM cell line compared to curcumin. These curcuminoid analogues gave higher cytotoxicity towards LN-18 human GBM cell line with more production of ROS and significantly severe DNA damage.

We first investigated these analogues’ cytotoxicity potential through MTT assay as these curcuminoid analogues are the newly synthesized novel compounds. Interestingly, our results found that these analogues were able to reduce the viability of LN-18 cells in dose-dependent manner with higher toxicity than curcumin. FLDP-5 and FLDP-8 curcuminoid analogues were synthesized through the addition of 4-piperidinone group to the curcumin skeleton. In relation to this, we hypothesized that these findings whereby the FLDP-5 and FLDP-8 curcuminoid analogues appeared to have higher potential than curcumin could be resulted from the added 4-piperidinone group. Our results were in agreement with previous studies by Eryanti et al. whereby their group synthesized analogues of curcumin with the addition of 4-piperidone group, namely (*N*-methyl-(3E,5E)-3,5-bis-(2-chlorobenzylidene)-4-piperidone and *N*-methyl-(3E,5E)-3,5-bis-(3-bromobenzylidene)-4-piperidone. They found that both analogues were able to inhibit proliferation in breast cancer cells (T47D) with IC_50_ values of 8 μM and 4 μM, respectively, following 24-h treatment. There were no reported curcumin-treated T47D cells in their study^36^. But then, an earlier study by Nejati-Koshki et al. reported that curcumin-induced cytotoxicity in T476D cell line after 24-h treatment at 28 μM^37^. Thus, we could see that analogues added with 4-piperidone group were able to induce higher cytotoxicity in the cancer cell line corresponding to our findings.

The selectivity of FLDP-5 and FLDP-8 curcuminoid analoges were verified by comparing the IC_50_ of both analogues against normal human cerebral microvascular endothelial cell (HBEC-5i). Both analogues depicted SI values in which higher than the ‘100’ baseline by several folds suggesting that the analogues were more selective towards cancerous cells than normal cells. However, further investigation should be carried out in future using normal glial and astrocytes cells to strongly confirm the selectivity of both analogues between brain cancerous and normal cells. We reported that curcumin have a high SI value with several folds higher compared to the baseline and also the analogues showing its selectivity. This results are in line with the previous study by Zanotto-Filho et al. that reported curcumin depicted a much higher value of IC_50_ in the normal cells compare to cancer cells indicating the cytototoxic effects of curcumin was selectively targeted at GBM^38^.

Limited and heterogeneous drug distribution across the BBB is a primary cause of treatment failure for otherwise promising novel drug treatments in GBM. Drug distribution through an intact BBB into the brain is a necessary first step in developing effective GBM therapeutics and must be a highlight concern in any clinical trial design of GBM^39^. In concern of that, we decided to investigate the permeability of these analogues across the BBB using two different online prediction tools. The predictions suggested that both FLDP-5 and FLDP-8 curcuminoid analogues were capable to cross the BBB in which an excellent output value of probability was observed in FLDP-5 curcuminoid analogue indicating its great potential. The prediction for curcumin with excellent probability of crossing the BBB was in agreement in previous studies that stated that curcumin was BBB permeable and was found in cerebrospinal fluid (CSF) due to its highly lipophilic property^40,41^.

Then, we further performed the DHE and DCFH-DA staining assays in order to investigate the role of ROS in inducing the cell death of LN-18 cells. Our findings suggested that curcumin, FLDP-5 and FLDP-8 curcuminoid analogues induced oxidative stress through the generation of ROS superoxide anion in a time-dependent manner. Significant ROS hydrogen peroxide production was also observed induced by these analogues at 6-h time treatment which differed with curcumin. Our curcumin data were consistent with a study reported by Yin research group where they found a non-significant effect of hydrogen peroxide on U87MG GBM cells^42^. In other previous studies, curcumin was confirmed to cause cell death in cancer cells through ROS production, where studies on gastric cancer cells and osteosarcoma cells reported that ROS induced apoptosis signal-regulating kinase 1 (ASK1)/ MAPK kinase (MKK) 4/ c-Jun N-terminal kinase (JNK) signaling pathway and mitochondrial cytochrome c/ caspase 3 apoptotic pathway respectively^43,44^ leading to apoptosis. In respect to that, further research should be carried out on FLDP-5 and FLDP-8 analogues to fully confirm the mechanism regarding involvement of ROS in inducing cell death in LN-18 cancer cell line.

ROS accumulation could directly damage DNA and cause oxidative lesions. In regards to that, our study demonstrated that FLDP-5 and FLDP-8 curcuminoid analogues induced DNA damage in LN-18 cells in a time-dependent manner. DNA damage was induced in accords with the elevated ROS production resulting in oxidative stress, as confirmed from the ROS assessment. The 6-h time-point for both treated FLDP-5 and FLDP-8 curcuminoid analogues appeared to have the most severe damages suggested that the severity could have resulted from the presence of both superoxide anion and hydrogen peroxide as demonstrated in the DHE and DCFH-DA staining experiment results. Particularly, hydrogen peroxide effects should be highlighted as the brain contains high amounts of unsaturated fatty acids (UFA), which are mainly found in the membrane phospholipids of the brain resulting in the brain to be especially vulnerable to damage from peroxides^45^. Detoxification of hydrogen peroxides usually occurs with presence of enzymes catalase and glutathione peroxidases (GPx), where the peroxides will be reduced into water and oxygen. But, several studies have demonstrated that catalase and GPx activity were greatly decreased in brain tumour causing the scavenging activity of peroxides to decline^46,47^. This explain as to the reason behind the severe damage that occurred in the 6-h time-point induced by both curcuminoid analogues.

In GBM, the spreading of this tumour is mainly due to its highly invasive nature and high rate of motility, leading to migration. Glioma cells can spread widely beyond the primary tumour and even pass into the contralateral hemisphere, making total surgical removal of GBM impossible^30,48^. Curcumin has been reported in various studies to have anti-migratory effects on GBM^49–51^. Therefore, in this study, we decided to investigate the ability of these analogues to inhibit the migration and invasion of LN-18 cells, and our findings revealed that the potential of these analogues in inhibiting migration and invasion in LN-18 cells. Curcumin reported to inhibit migration and invasion in GBM in in-vitro studies as well as in in-vivo models of GBM cells through various pathways such as regulation of proteins MMP-2/9, fascin expression, SHH/GLI1 pathway and miRNA^51–53^. So, a thorough research investigating the pathways that caused the inhibition of migration and invasion which may be through regulation of MMP-2/9 as illustrated in Fig. 11 of these novel analogues should be conducted as it could greatly strengthen the understanding of anti-migratory effects of these analogues.

Moreover, our study also found that FLDP-5 and FLDP-8 curcuminoid analogues were able to induce cell cycle arrest through inhibition at the S phase in LN-18 cells. The inhibition at the S phase suggested that the cells may be prevented from progressing to G_2_/M phase, thus preventing the cells to undergo mitosis^51^. Our results demonstrated a differed findings of cell cycle arrest between curcuminoid analogues and curcumin in which curcumin showed inhibition at G_2_/M phase. Our curcumin data were in agreement with previous studies that reported curcumin significantly inhibits GBM cell growth and proliferation via the suppression of cell cycle progression in different human glioma cell lines at G_2_/M phase^54–56^. Curcumin caused G_2_/M cell cycle arrest in a p53-dependent manner, according to Liu et al. In fact, curcumin increases p53 protein levels in U251 glioma cells, followed by induction of CDK inhibitor /cell-cycle regulator p21 and tumor suppressor ING4, thus resulting in cell cycle arrest. Another study found that U251-treated cells are inhibited in the G2/M phase due to increased expression of the tumour suppressor death-associated protein kinase 1 (DAPK1), which is accompanied by suppression of the NF-B and STAT3 pathways, as well as caspase 3 activation^55^. Anyhow, the pathway that induced by these novel analogues should be studied more in depth as it gives a new perspective contradict with previous curcumin data.

**Figure 9:**
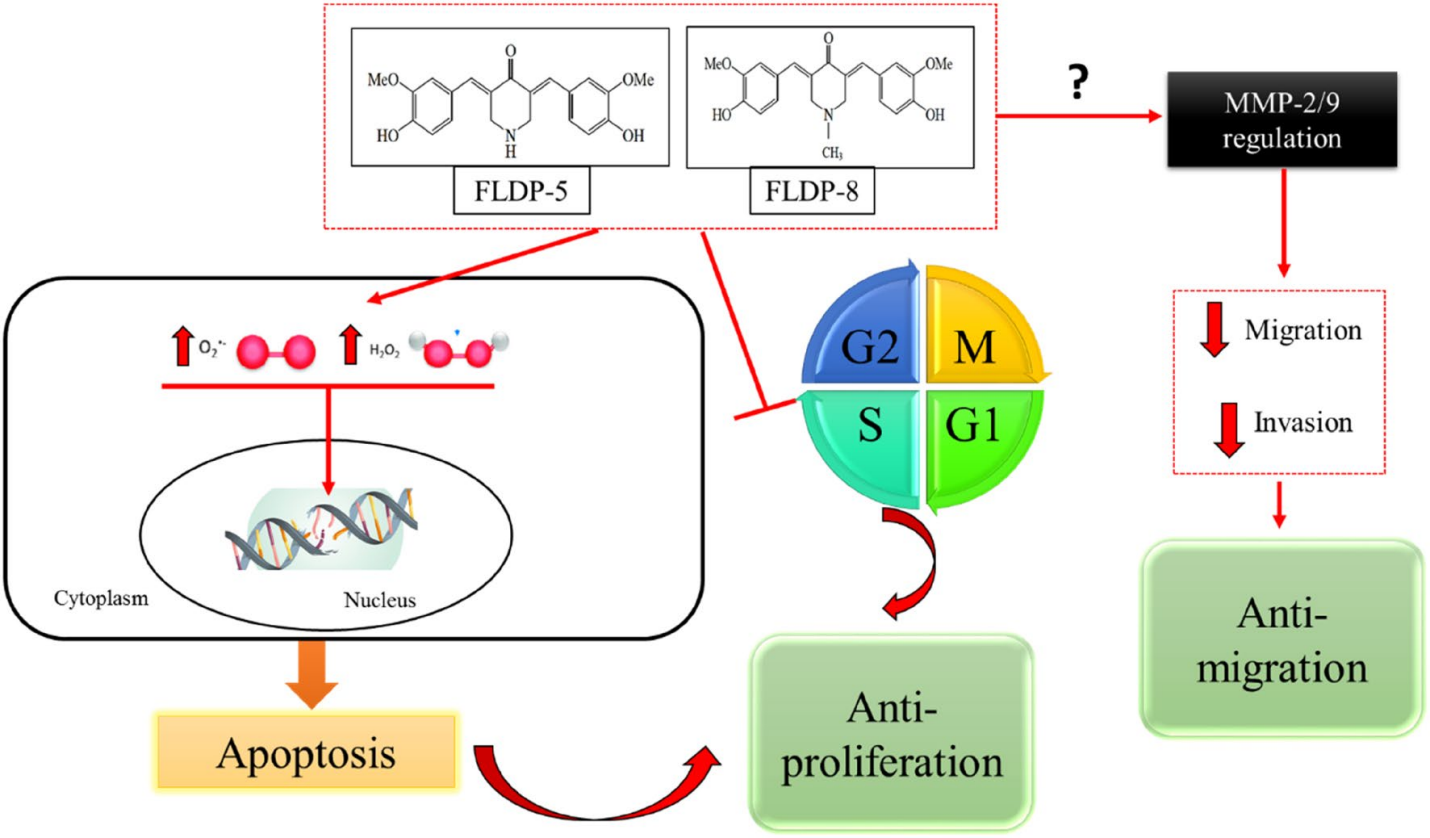
Schematic representation of FLDP-5 and FLDP-8 curcuminoid analogues induced anti-proliferative and anti-migratory effects in LN-18 human GBM cells. Curcuminoid analogues (FLDP-5 and FLDP-8) induced DNA damage through oxidative stress with the increase in the production of intracellular ROS. The analogues also induced S-phase cell cycle arrest leading to anti-proliferative effect on LN-18 cells. Moreover, FLDP-5 and FLDP-8 curcuminoid analogues potentiate anti-migratory effect on LN-18 cells through inhibition in the migration and invasion of this cell line.

In recent years, a significant research effort has also been focused on synthesizing new panel analogues of curcumin in overcoming its drawbacks. The low cancer-killing potency of curcumin, its multiple biological effects, and its low bioavailability were the major factors curcumin analogues with similar safety profiles but increased anti-cancer activity and solubility were designed. EF24 (diphenyl difluoroketone) is one such analogue that recently gained a high interest as this analogue exhibits potent anti-cancer activity in colon and gastric cancer^57^. Nonetheless, taken together, our results have proven that these analogues possessed potent anti-cancer activity as summarized in the schematic representation in Fig. 9, making it a worthwhile study to pursue.

## Conclusion

Overall, our findings elucidate the potential of FLDP-5 and FLDP-8 curcuminoid analogues in LN-18 human GBM cells. This study has demonstrated that these curcuminoid analogues exhibited anti-cancer effects with anti-proliferative, anti-migratory and BBB permeable properties in GBM with higher potency compared to curcumin. However, further investigation into the underlying mechanism that causes cell death should be carried out as it could greatly enhance the understanding of the anti-cancer role of these compounds.

## Methods

### Reagents

Dulbecco’s Modified Eagle’s Medium (DMEM) Medium from *Gibco Invitrogen*, USA; penicillin/streptomycin from *Nacalai Tesque Inc*., Kyoto, Japan; fetal bovine serum (FBS) from *Tico Europe,* Amstelveen, Netherlands; phosphate buffer saline (PBS), (3-(4,5-dimetiltiazol-2-il)-2,5-difenil tetrazolium bromide) (MTT) from *Sigma-Aldrich,* UK; dimethylsulphuxide (DMSO) and hydrochloric acid (HCl) form *Fisher Scientific,* UK; sodium hydrogen carbonate (NaHCO_3_) and potassium hydrogen phosphate (KH_2_PO_4_) from *Systerm,* Malaysia; sodium chloride (NaCl) and sodium dihydroxide (NaOH) from *EMSURE,* Jerman; disodium hydrogen phosphate (Na_2-h_PO_4_) from *HmbG Chemicals,* German; potassium chloride (KCl) from *J.T Baker,* USA; dihidroethidium (DHE) stain and dichlorofluorescin-diacetate (DCFH-DA) stain from *Eugene*; Disodium ethylenediaminetetraacetate dihydrate (Na_2_EDTA), low-melting point agarose (LMA) and normal melting agarose (NMA) from *Sigma-Aldrich*, St. Louis, MO, USA; Tris from *Bio-Rad Laboratories*, Hercules, CA, USA; 70% alcohol and distilled water from the FSK laboratory.

### Test compounds

Compounds 4-Peperidinone,3,5-bis[(4-hydroxy-3-methoxyphenyl) methylene]-,(3E,5E) (FLDP-5) and 4-Peperidinone, 3,5-bis[(4-hydroxy-3-methoxyphenyl) methylene]-1-Methyl(3E,5E) (FLDP-8) (Fig. 1) were synthesized and contributed by Dr. Lam Kok Wai from Centre for Drug and Herbal Development, Faculty of Pharmacy, Universiti Kebangsaan Malaysia (Kuala Lumpur, Malaysia). Curcumin and hydroquinone were purchased from *Sigma-Aldrich* (St. Louis, MO, USA). Stock solutions of FLDP-5 and hydroquinone were prepared at 50 mM, while stock solutions for FLDP-8 and curcumin were prepared at 25 mM. All compounds were dissolved in solvent dimethyl sulfoxide (DMSO). Treatment for all compounds on LN-18 cells was performed in dark condition due to the compounds’ photosensitive characteristics.

### Cell culture

LN-18 human GBM cells were established in 1976 from cells taken from a patient with a right temporal lobe glioma whereas HBEC-5i cells were established in 1994 which were derived from small fragments of human cerebral cortex obtained from patients who had died of various causes and were devoid of any pathologic abnormalities. Both cell lines were obtained from American Type Culture Collection (ATCC). The culture medium used throughout these experiments was Dulbecco’s Modified Eagle’s Medium (DMEM) medium (GIBCO) for both cell lines supplemented with fetal bovine serum (FBS) (5% for LN-18 cells and 10% for HBEC-5i cells) and 1% penicillin/streptomycin. Both LN-18 and HBEC-5i cells were used between passages 3–12 for the experiments and maintained at 37 °C and 5% CO_2_.

### MTT cytotoxicity assay

The cytotoxic effects of curcumin and the curcuminoid analogues (FLDP-5 and FLDP-8) were assessed as previously described^58^. Briefly, LN-18 cells were seeded in a 96-well plate at a density of 5 × 10^4^ cells per well in a volume of 200 µL and were treated with curcuminoid analogues (FLDP-5 and FLDP-8), curcumin and hydroquinone (positive control) with respective concentrations. The treated cells were incubated for 24-h under 5% CO_2_ at 37 °C. After 24-h incubation, 20 µL of 5 mg/mL MTT was added to each treated cells and further incubated for 4-h at 37 °C. MTT salt was reduced forming a purple-coloured crystal formazan by the active viable cells with dehydrogenase enzyme. Then, 180 µL of the medium was discarded from the treated cells before adding 180 µL of dimethyl sulfoxide (DMSO) to solubilize the crystal formazan, respectively. The plate was further incubated for 15 min to completely dissolve the crystal formazan, followed by gentle resuspension for each well. The cytotoxic effects of each compound were detected by measuring the absorbance of each well at 570 nm using iMark™ microplate reader (*Bio-Rad Laboratories*, Hercules, CA, USA). The inhibitory concentration that kills 50% of the cell population (IC_50_) was obtained from the plotted of each compounds concentrations versus the percentage of the cell viability.

### Selectivity index

The selective index (SI) of the compounds were calculated according to the equation as previously established^28^. The calculation is done in order to determine the degree of selectivity of the compound tested against cancerous cells, in which values larger than “100” indicates the compound is selective toward cancerous cells and confers minimal toxicity in normal non-malignant cells. In this study, the SI values were determined by IC_50_ of normal human cerebral microvascular endothelial cell (HBEC-5i) divided by IC_50_ of LN-18 cells for each compound. It was simplified as follows:

$${\text{Selective Index }}(SI) = \frac{{IC_{{50}} {\text{ of}}\;{\text{ HBEC}} - 5i}}{{IC_{{50}} {\text{ of }}\;{\text{LN}} - 18}}{\text{ }} \times 100\%$$

### Blood–brain barrier (BBB) and ADMET prediction

PubChem database was applied to get the smiles structures of FLDP-5 and FLDP-8 curcumioid analogues, and was further used for the BBB and ADMET prediction using two different online platforms, AlzPlatform (www.cbligand.org/AD/) and ADMETlab 2.0 (https://admetmesh.scbdd.com/)^59–62^. AlzPlatform was built by using the support vector machine (SVM) and LiCABEDS algorithms on PubChem fingerprint from 1593 reported compounds. By entering the smiles structures of the compounds, the online predictor calculated the BBB score, which showed whether a compound could pass the blood–brain barrier (BBB +) or not (BBB−). ADMETlab 2.0 which is an integrated online platform, was used to computationally predict the pharmacokinetic properties of compounds such as absorption, distribution, metabolism, excretion, and toxicity (ADMET).

### Reactive oxygen species assessment

The level of reactive oxygen species (ROS), specifically for superoxide anion and hydrogen peroxide were assessed as previously described^63,64^. Briefly, the treated LN-18 cells were administered at different time-point intervals before being harvested. The treated LN-18 cells were then collected by centrifugation at 220 ×  *g* for 5 min. After the supernatant was discarded, the pellet was resuspended with 1 mL of fresh pre-warmed FBS-free DMEM media and with the addition of 1 µL of 10 mM DHE and 10 mM DCFH-DA stains. The cells suspension with DHE and DCFH-DA staining were incubated at 37 °C for 30 min. After the incubation period, the cells were centrifuged at 220 × g for 5 min. Then, the cells were washed with 1 mL chilled PBS, and the supernatant was discarded, followed by resuspension of the pellet by 500 µL of ice-cold PBS. The stained cells were transferred to flow cytometric analysis tubes and analyzed using FACSCanto II flow cytometer (BD Bioscience, USA) on 10,000 cells.

### Alkaline comet assay

As previously described, the alkaline comet assay was performed to access DNA damage induced by curcumin and the curcuminoid analogues (FLDP-5 and FLDP-8)^65,66^. Treated LN-18 cells were harvested and washed twice with Ca2 + , Mg2 + -free PBS. Cell pellets were then mixed thoroughly with 80 µL of 0.6% low melting point agarose (LMA) and laid on hardened 0.6% normal melting agarose (NMA). The agarose was allowed to solidify and subsequently placed in a chilled lysis buffer (2.5 M NaCl, 100 Mm EDTA, 10 mM Tris, and 1% Triton-X) for lysis to occur. Slides were then incubated in an electrophoresis buffer (0.3 N NaOH, 1 Mm EDTA) for 20 min to facilitate DNA unwinding. Electrophoresis was performed under 25 V, 300 Ma for 20 min. Subsequently, slides were rinsed with neutralizing buffer (400 Mm Tris) thrice prior to staining with 50 µg/mL ethidium bromide solution. The slides were observed under Olympus BX51 fluorescence microscope (Olympus, Japan) equipped with 590 nm filter. The tail moment, the product of tail length and fraction of total DNA in the tail, was assessed using Comet Score™ software (TriTek Corp, Sumerduck, VA, USA) on 50 cells per slide.

### Scratch/wound-healing assay

A monolayer wound healing assay was performed following the protocol from previous studies with slight modifications^67,68^. LN-18 cells were seeded at a density of 2 × 10^5^ cells per well in a 12-well plate (*Nest Biotechnology*, Jiangsu, China). After reaching 90% confluency, the cells monolayer were then scraped in a straight line creating a “scratch” using a sterile 200 μL pipette tip. The cells were then washed with PBS before taking photographs of the scratched area using a camera attached to an inverted phase contrast microscope (Olympus, Japan). The scratch area were photographed, and the location on the plate was noted. The cells were then treated with FLDP-5 curcuminoid analogue (1.25 and 2.5 µM), FLDP-8 curcuminoid analogue (2.5 and 5 µM) and curcumin (12.5 and 25 µM). Untreated cells were used as a control for the experiment. Cells were further incubated for 24-h and 48-h before the same area were photographed, and the cells migration area was measured using Image J software before the percentage of wound closure was calculated.

### Boyden chamber invasion assay

The principle of this assay is based on two medium containing chambers separated by a porous membrane through which cells transmigrate^69^. This assay was conducted following the protocol provided by QCM™ Collagen Cell Invasion Assay, 24-well (8 μm), Colorimetric kit purchased from *Merck*, Germany. Generally, LN-18 cells were starved for 24-h prior to assay in serum-free DMEM medium. Then, 250 μL of harvested cell suspension with concentration of 7 × 10^5^ cells/mL in chemo-attractant free media was added to the insert/upper chamber of the well containing collagen-coated membrane. After that, 500 μL of DMEM containing respective compounds treatment was added to the bottom chamber of the well. After 24-h incubation, the insert coated with the membrane will be fixed and stained with 400 μL Cell Stain. A cotton-tipped swab was used to remove the non-invading cells/collagen layer from the interior of the insert. The stained insert was transferred to a new well containing 200 μL of Extraction Buffer, and 100 μL of the dye mixture was transferred into a 96-well plate. The Optical Density (OD) of invaded cells were measured using iMark™ microplate reader (*Bio-Rad Laboratories*, Hercules, CA, USA) at 560 nm.

### Cell cycle analysis

Cell cycle distribution was determined following protocol as previously described^70^. Cells were seeded at 2 × 10^5^ cells per well in a 6-well plate before being treated with curcuminoid analogues (FLDP-5 and FLDP-8) and curcumin for 24-h. The treated cells will be harvested and washed with chilled PBS before being fixed with 70% alcohol for at least overnight before staining. After fixing, cells will be washed with PBS and later stained with PI/RNase staining buffer (500 μL) (BD Bioscience) for 15 min at room temperature. Stained cells will then be analysed by using FACSCanto II flow cytometer (*BD Bioscience*, USA) on 20,000 cells, and the content of DNA will be determined by using ModFit LT™ software (Verity Software House).

### Statistical analysis

The data are expressed as the mean ± standard error of mean (S.E.M.) from at least three independent experiments. The statistical significance was evaluated using one-way ANOVA with the Dunnet post hoc test to assess significance difference with negative control (NEG) or Tukey post hoc test to determine the significance of differences between multiple treatment groups. Differences were considered statistically significant with a probability level of *p* < 0.05.

## Supplementary Information

Supplementary Information.

## Data Availability

All data generated or analyzed during this study are included in this published article (and its supplementary information files). The data are available from the corresponding author upon request.
