# Supplementary material for: Curcumin piperidone derivatives induce anti-proliferative and anti-migratory effects in LN-18 human glioblastoma cells
Source: Sci Rep. 2022 Jul 30;12:13131. doi: 10.1038/s41598-022-16274-4 (PMC9338982; doi:10.1038/s41598-022-16274-4)
Supplement: Supplementary file 1 — Supplementary Information. [file 41598_2022_16274_MOESM1_ESM.docx]

**Supplementary Information**

**Title:** Curcumin piperidone derivatives induce anti-proliferative and anti-migratory effects in LN-18 human glioblastoma cells

**Authors:**

Nur Syahirah Che Razali^1^, Kok Wai Lam^3^, Nor Fadilah Rajab^2^, A Rahman A Jamal^4^, Nurul Farahana Kamaluddin^1^, Kok Meng Chan^1, 5*^

**Authors Affiliation:**

^1^Center for Toxicology and Health Risk Studies, Faculty of Health Sciences, Universiti Kebangsaan Malaysia, 50300 Kuala Lumpur, Malaysia

^2^Center for Health Ageing and Wellness Studies, Faculty of Health Sciences, Universiti Kebangsaan Malaysia, 50300 Kuala Lumpur, Malaysia

^3^Centre for Drug and Herbal Development, Faculty of Pharmacy, Universiti Kebangsaan Malaysia, 50300 Kuala Lumpur, Malaysia

^4^UKM Medical Molecular Biology Institute, UKM Medical Centre, 56000 Cheras, Malaysia

^5^Institute for Environmental and Development (LESTARI), UKM, 43600 Bangi, Selangor, Malaysia

*Email: chan@ukm.edu.my


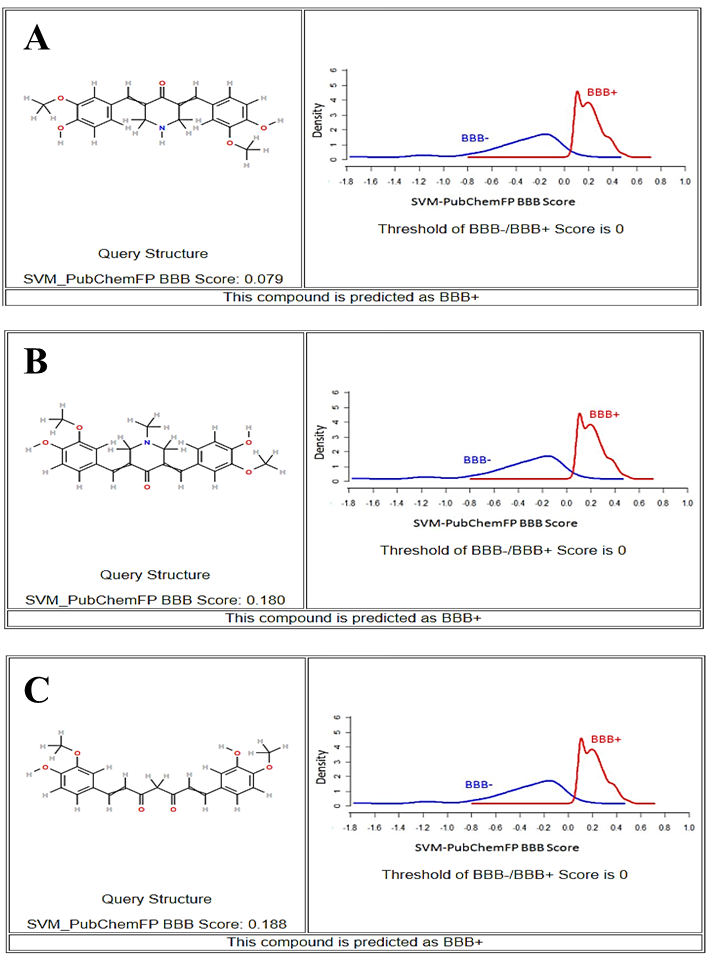


**Figure. S1. The prediction of the probability of the analogues penetrating BBB by the online platform AlzPlatform (www.cbligand.org/AD/).** The online predictor gave the corresponding calculation BBB score, which indicates whether a compound can cross the blood−brain barrier (BBB+) or not (BBB−). (**A**) The analysis showed that FLDP-5 curcuminoid analogue had a positive BBB score, which is higher than the threshold of BBB-/BBB+ score, implying that this analogue is penetrable to BBB. (**B**) FLDP-8 curcuminoid analogue had a positive BBB score, which is higher than the threshold of BBB-/BBB+ score, implying that this analogue is penetrable to BBB. (**C**) Curcumin had a positive BBB score, which is higher than the threshold of BBB-/BBB+ score, implying that curcumin is penetrable to BBB.

**Table S1. The prediction of the probability of the analogues penetrating BBB and bioavailability by the online platform ADMETlab 2.0 (https://admetmesh.scbdd.com/)**

| **Model** | **Result of FLDP-5** | **Probability** | **Result of FLDP-8** | **Probability** | **Result of curcumin** | **Probability** |
| --- | --- | --- | --- | --- | --- | --- |
| **Absorption** | | | | | | |
| Caco-2 Permeability | Permeable (Excellent) | -5.044 | Permeable (Excellent) | -4.929 | Permeable (Excellent) | -4.831 |
| MDCK Permeability | Permeable (Excellent) | 1.5e-05 | Permeable (Excellent) | 1.8e-05 | Permeable (Excellent) | 1.4e-05 |
| HIA | HIA+ (Excellent) | 0.171 | HIA+ (Medium) | 0.613 | HIA+ (Excellent) | 0.013 |
| F_30%_ | F_30%_ + (Excellent) | 0.057 | F_30%_ + (Excellent) | 0.021 | F_30%_ + (Poor) | 0.798 |
| **Distribution** | | | | | | |
| Blood-brain barrier | BBB+ (Excellent) | 0.029 | BBB+ (Medium) | 0.38 | BBB+ (Excellent) | 0.155 |
